# Biofilm may not be Necessary for the Epidemic Spread of *Acinetobacter baumannii*

**DOI:** 10.1038/srep32066

**Published:** 2016-08-25

**Authors:** Yuan Hu, Lihua He, Xiaoxia Tao, Fanliang Meng, Jianzhong Zhang

**Affiliations:** 1 State Key Laboratory of Infectious Disease Prevention and Control, Collaborative Innovation Center for Diagnosis and Treatment of Infectious Diseases, Chinese Center for Disease Control and Prevention, Beijing, 102206, China; 2 National Institute for Communicable Disease Control and Prevention, Chinese Center for Disease Control and Prevention, Beijing, 102206, China

## Abstract

Biofilm is recognized as a contributing factor to the capacity of *Acinetobacter baumannii* to persist and prosper in medical settings, but it is still unknown whether biofilms contribute to the spread of *A. baumannii*. In this study, the biofilm formation of 114 clinical *A. baumannii* isolates and 32 non-*baumannii Acinetobacter* isolates was investigated using a microtiter plate assay. The clonal relationships among *A. baumannii* isolates were assessed using pulsed-field gel electrophoresis and multilocus sequence typing, and one major outbreak clone and 5 other epidemic clones were identified. Compared with the epidemic or outbreak *A. baumannii* isolates, the sporadic isolates had significantly higher biofilm formation, but no significant difference was observed between the sporadic *A. baumannii* isolates and the non-*baumannii Acinetobacter* isolates, suggesting that biofilm is not important for the epidemic spread of *A. baumannii*. Of the multidrug-resistant (MDR) *A. baumannii* isolates in this study, 95.7% were assigned to international clone 2 (IC2) and showed significantly lower biofilm formations than the other isolates, suggesting that biofilm did not contribute to the high success of IC2. These findings have increased our understanding of the potential relationship between biofilm formation and the epidemic capacity of *A. baumannii*.

*Acinetobacter* spp. are recognized as important opportunistic Gram-negative pathogens that are found mainly in immunocompromised patients. However, great diversity exists in the clinical importance of the various *Acinetobacter* species, with some being dominant as human pathogens and others merely acting as colonizing or environmental organisms^1^. Some *Acinetobacter* species are highly successful in their capacity to cause outbreaks or to develop antibiotic resistance, among which *A. baumannii* is the most clinically important species, with the greatest number of healthcare-related outbreaks and reports of multidrug resistance^2^. The number of multidrug-resistant (MDR) *A. baumannii* outbreaks is currently increasing worldwide. Many of the genotypes involved belong to three predominant clones (international clones, ICs), of which IC2 is often MDR and is predominant in outbreaks of *A. baumannii* infection worldwide^3^.

Thus far, the attributes that render some *Acinetobacter* species or some clones (lineages) more adept at causing human outbreaks and disease are poorly understood. Two key factors contributing to the significant and ubiquitous dissemination of *A. baumannii* in hospitals are the extent of its antimicrobial resistance and its environmental resilience, which were proposed to be due to the capacity of this bacterial pathogen to form biofilms on abiotic surfaces^4^,^5^,^6^,^7^. However, great variation exists in the biofilm formation capacity of *A. baumannii* clinical isolates^8^. Whether the variation in biofilm formation among strains determines their epidemic differences is still unknown. In this study, the biofilm formations were investigated for a large set of *A. baumannii* and non-*baumannii Acinetobacter* (non-AB) isolates that differed in terms of their epidemicity and drug resistant level.

## Results

### Comparison of biofilm formation in *A. baumannii* and non-AB isolates

The biofilm formation capacities of 114 *A. baumannii* isolates and 32 non-AB isolates were evaluated. The characteristics of the isolates are shown in Table 1. The ratio between the average optical density (OD) of the stained biofilm and the cut-off OD value (ODc) was selected to represent the biofilm formation of each isolate. Biofilm was detected in 36% (41/114) of the clinical *A. baumannii* isolates and 81.3% (26/32) of the non-AB isolates. Of the *A. baumannii* biofilm-positive isolates, 19.5% (8/41) were strong biofilm producers. In contrast, 34.6% (9/26) of the non-AB biofilm-positive isolates were strong biofilm producers, as shown in Table 2. The 32 clinical non-AB isolates showed higher biofilm formation than the 114 clinical *A. baumannii* isolates (Fisher’s exact test, P < 0.0001). Of the non-AB isolates, 75% were non-MDR (Table 1), so we compared the biofilm formation capacities of non-AB isolates to the non-MDR *A. baumannii* isolates, and no significant difference was observed between them (Table 2). The individual biofilm formation capacities are outlined in supplementary Table S1.

### Pulsed-field gel electrophoresis (PFGE) analysis of the *A. baumannii* isolates

**Figure 1:**
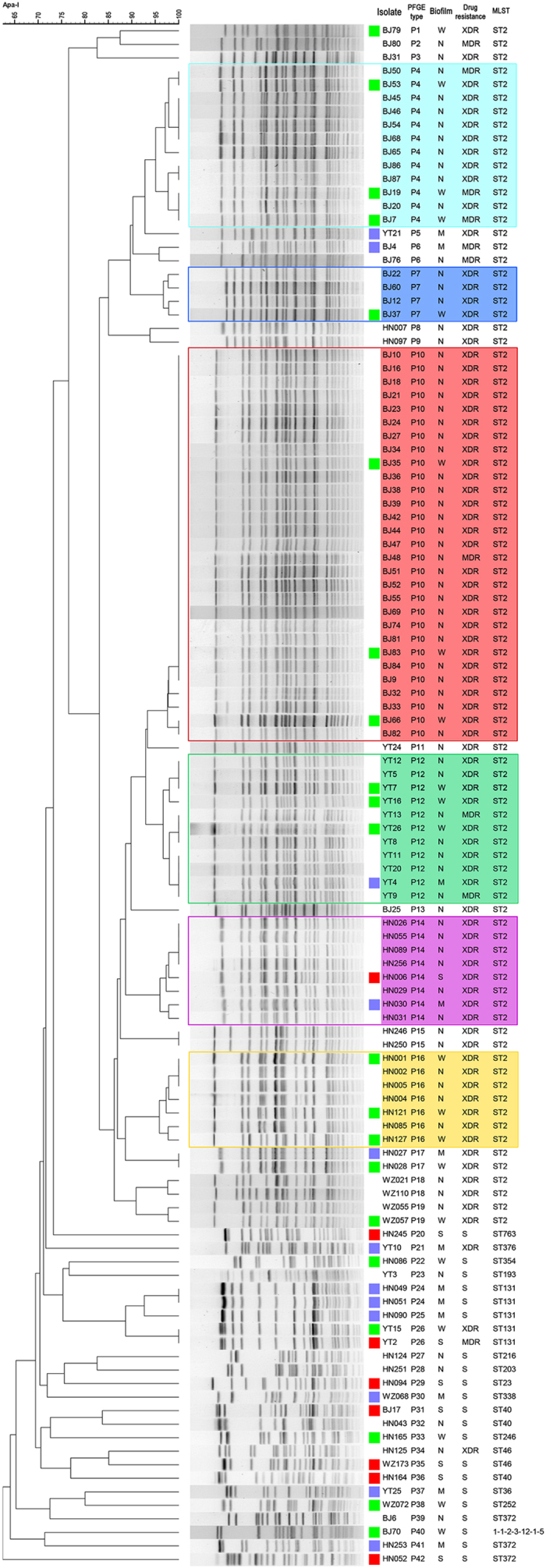
Biofilm formation of the 114 clinical *A. baumannii* isolates and the related PFGE typing. The dendrogram of the PFGE patterns is shown on the left. The related results of biofilm formation and antimicrobial susceptibility are provided for direct comparison. Weak biofilm producer (W), moderate biofilm producer (M) and strong biofilm producer (S) are marked by 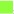, 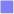 and 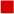, respectively, on the right of the PFGE profile. Isolates belonging to outbreak and epidemic clones are marked with coloured backgrounds.

The clonal relationships between *A. baumannii* isolates were assessed using pulsed-field gel electrophoresis (PFGE). The 114 *A. baumannii* isolates tested herein for biofilm formation represented 42 unique PFGE types (P1~P42), as shown in Fig. 1 and Table 3. All isolates sharing the same PFGE type were isolated from the same hospital (Table 3). Compared with the MDR isolates, a higher genetic diversity was revealed in the non-MDR isolates (Fig. 1). We define an isolate as being epidemic if at least two other isolates isolated from the same hospital during the study period exhibited the same PFGE profile (with ≥95% similarity in their banding patterns). Isolates clustering according to these features were regarded as epidemic clones, while all other isolates were considered sporadic. A total of 6 epidemic clones were revealed (P4, P10, P7, P12, P14, P16), which covered 62.3% of the tested *A. baumannii* isolates, as shown in Fig. 1 and Table 3. However, one of the epidemic clones (P10) was responsible for a major outbreak involving 29 patients and this clone was termed outbreak clone. All the epidemic isolates (including the outbreak isolates) were MDR.

### Multilocus sequence typing (MLST) analysis of the *A. baumannii* isolates

To identify the evolutionary lineages, all the *A. baumannii* isolates were analysed by MLST and clustered into 17 sequence types (STs), as shown in Table 4. All the isolates sharing the same PFGE type were also assigned to the same ST (Table 3). A total of 89 (78%) *A. baumannii* isolates, representing PFGE types P1 to P19 isolates shown in Fig. 1, were assigned to ST2 of the IC2 (Table 4), which covered 95.7% of the MDR isolates, including all the epidemic isolates. Of the IC2 isolates, 93.3% showed weak biofilm forming capacities, of which 75.3% (67/89) were non-biofilm producers and 18% (16/89) were weak biofilm producers (Table 4). Only one IC2 isolate (HN006) was a strong biofilm producer (mean OD/ODc = 13.24, Table S1), which showed a similar but unique PFGE profile within the P14 clone, which differed by an additional band (Fig. 1). Thus, this stronger IC2 biofilm producer was not widely spread during our study period. The other 5 IC2 moderate biofilm producers originated from 3 hospitals and were assigned to 5 PFGE types (Table 3). Only two of these IC2 moderate biofilm producers belonged to epidemic clones.

Compared with the IC2 isolates, the other isolates (25 isolates representing 16 STs) showed significantly higher biofilm formation (biofilm-positive rate of 24.7% vs. 76%, Table 4, Fisher’s exact test, P < 0.0001).

### Comparison of biofilm formation capacities between outbreak and epidemic *A. baumannii* isolates

During our study period, no *A. baumannii* infection outbreak was identified except for one hospital. A total of 54 isolates isolated during this outbreak period were used in this study, which were typed into 11 PFGE types (P4, P7, P10, P1~3, P6, P13, P31, P40, P39, Table 3). Among them, the P10 clone which covered 29 isolates was identified to be responsible for this outbreak. To determine whether biofilm was one possible reason for this outbreak, we compared the biofilm formation of the P10 clone with other epidemic clones that did not cause higher isolation rates than the exception. Contrary to our expectation, although there was no significant difference, a lower biofilm-positive rate was observed for the P10 clone (10.3% vs. 31%), as shown in Table 2. Therefore, biofilm formation did not contribute to the high isolation of this outbreak clone.

### Comparison of biofilm formation capacities between epidemic and sporadic*A. baumannii* isolates

A total of 43 *A. baumannii* isolates representing 36 unique PFGE types were identified as sporadic isolates, which showed significantly higher biofilm-forming capacity than the epidemic isolates (biofilm-positive rate of 58.1% vs. 31%, Fisher’s exact test, P = 0.0047, Table 2). Of the biofilm-negative sporadic isolates, 72.2% (13/18) were MDR; therefore, a sub-classification according to drug resistance was performed. For the biofilm-positive sporadic *A. baumannii* isolates, the OD/ODc ratios ranged from 1.03 to 24.08 for the non-MDR sporadic clones and from 1.06 to 4.9 for the MDR sporadic clones (Table S1). Although a higher biofilm-positive rate was observed in non-MDR sporadic isolates than in the MDR sporadic isolates (76.2% vs. 41%), no significant difference was observed between them (Table 2). However, a significant difference was observed between the non-MDR sporadic isolates and the epidemic isolates (Table 2).

Taking into account that we could not exclude the possibility that the MDR sporadic isolates would cause an epidemic at another time or in another hospital, we compared the biofilm formation capacities between all the MDR and non-MDR isolates. A significantly higher biofilm formation capacity was observed in the non-MDR isolates (biofilm-positive rate of 76.2% vs. 26.9%, Fisher’s exact test, P < 0.0001). However, no significant difference was noted among the three MDR isolate groups (outbreak, epidemic and MDR sporadic, Table 2).

## Discussion

There have been some reports on the variations in biofilm formation capacity among clinical isolates of *A. baumannii*^9^,^10^,^11^,^12^, but the quantitative differences in biofilm formation among clinical isolates, in association with the epidemic capacity of strains, have been poorly investigated thus far. In this study, the biofilm formation capacity was evaluated in a large set of well-described clinical *Acinetobacter* isolates. Contrary to our expectation, the non-AB isolates showed a higher biofilm formation than did the *A. baumannii* isolates. Among the *A. baumannii* isolates, the non-MDR ones showed a higher biofilm formation capacity than the MDR isolates, including all the epidemic clones. Even when comparing the non-AB isolates to only the non-MDR *A. baumannii* isolates, there was still no higher biofilm formation observed for *A. baumannii*, suggesting that biofilm-forming capacity could not explain the clinical success of *A. baumannii.* For *A. baumannii* isolates, the strong biofilm producers were less frequently resistant to antibiotics and seemed to be less epidemic, suggesting that biofilm is not necessary for the epidemic spread of *A. baumannii*.

A high proportion (95.7%) of the MDR *A. baumannii* isolates used in this study were assigned to IC2 by MLST, which agreed with previous reports that multidrug resistance is often associated with isolates that belong to international clones^13^,^14^,^15^. Distinct genetic diversity among the IC2 isolates was revealed by PFGE, with only some of those isolates demonstrating epidemicity during our study period; no significant difference was observed between the epidemic and sporadic IC2 isolates. Although the other ST lineages revealed in this study were not as successful as the IC2, which is widely spread worldwide and include strains that are usually MDR and associated with outbreaks, a significantly higher biofilm formation capacity was observed for non-IC2 than for the IC2 isolates, suggesting that biofilm does not contribute to the success of IC2.

It remains an open question whether *A. baumannii* were first to develop MDR and then lost their biofilm-forming capability or whether weak biofilm isolates were more prone to develop MDR, promoted by survival pressure. A recent study of isogenic mutants from a susceptible *A. baumannii* clinical isolate demonstrated the overproduction of resistance-nodulation-cell division (RND)-type efflux systems, AdeABC and AdeIJK, which pump out a wide range of antimicrobial compounds and are associated with multidrug resistance in *A. baumannii*^16^, resulting in the acquisition of antibiotic resistance and decreased biofilm formation^17^. This observation demonstrated the hypothesis that *A. baumannii* lost their biofilm-forming capability after developing MDR, but this model still needs further confirmation. However, the mechanism maybe more complicated than our speculation and cannot be answered with only one hypothesis. Whatever the truth is, we can speculate that compared with the MDR isolates, the non-MDR *A. baumannii* isolates are easily cleared after infection, so the capacity to grow as a biofilm may play a more important role in their persistence. Therefore, although high genetic diversity was revealed in the non-MDR isolates, a high proportion of them still maintained strong biofilm-forming capabilities.

In conclusion, the sporadic *A. baumannii* isolates have significantly greater biofilm-forming capabilities than the outbreak and epidemic *A. baumannii* isolates, but they showed biofilm formation capabilities that were similar to the other *Acinetobacter* species, suggesting that biofilm formation could not explain the clinical success of *A. baumannii* and is not important for the epidemic spread of *A. baumannii*. The IC2 isolates showed significantly lower biofilm formation capacity than other isolates, suggesting that biofilm did not contribute to the success of IC2. These findings have refreshed our understanding of the relationship between biofilm formation and *A. baumannii* epidemic capacity and may serve as caveats for future studies to understand the transmission of this pathogen.

## Materials and Methods

### Bacterial strains

A collection of 114 well-characterized *A. baumannii* isolates and 32 non-AB isolates were used (4 *A. bereziniae* isolates, 8 *A. nosocomialis* isolates, 13 *A. pittii* isolates, and 7 *A. junii* isolates, Table 1). The *A. baumannii* isolates included in the present study were from a collection of clinical isolates recovered during epidemiological surveys (from 4 Chinese cities, one hospital per city, not more than 2 months). All isolates were identified by matrix-assisted laser desorption/ionization time-of-flight (MALDI TOF) mass spectrometry^18^ and were verified using sequence analysis of the 16S-23S ribosomal DNA intergenic spacer^19^.

The antimicrobial susceptibilities of the tested *Acinetobacter* isolates to 11 antimicrobials were performed using an Etest on Mueller-Hinton agar. If a strain was resistant to at least three classes of antimicrobial agents, including all penicillins and cephalosporins (including inhibitor combinations), fluoroquinolones, and aminoglycosides, then that strain was called MDR. An MDR strain also resistant to carbapenems was called extensively drug-resistant (XDR)^20^.

### PFGE and MLST

The clonal relationships between *A. baumannii* isolates were assessed using PFGE, as previously described^21^. The PFGE patterns were analysed with BioNumerics software (Applied Maths) using the Dice coefficient and the unweighted-pair group method with average linkages (UPGMA), a 1.5% tolerance limit and 1.5% optimization. MLST was performed according to the published Pasteur protocols^22^.

### Biofilm formation

Biofilm formation was examined by the semi-quantitative determination of biofilm formation in a 96-well microtiter plate assay, as previously described^12^. Cultures were inoculated in Luria-Bertani broth (LB) and adjusted to an optical density at 600 nm of ~0.1. Each well of sterile 96-well polystyrene microtiter plates was filled with 200 μL of bacterial suspension. Wells containing only the medium were used as negative controls. After static incubation at 37 °C for 24 h, the plates were washed gently three times with phosphate-buffered saline to remove unattached bacteria, air-dried and stained with 0.1% crystal violet solution for 20 min, then scanned at 570 nm to determine the OD of the stained biofilms. The same protocol was followed to quantify the biofilm after prolonged incubation for 48 and 72 hours, and the maximum values obtained under the three incubation times were selected to represent the biofilm-forming capacity to avoid variations due to differences in biofilm formation rate. All assays were performed in triplicate at three independent time-points using fresh samples each time. The ODc was defined as three standard deviations above the mean OD of the negative control^23^. Each isolate was classified as follows^23^: non-biofilm producer (N): OD ≤ ODc; weak biofilm producer (W): ODc < OD ≤ 2 × ODc; moderate biofilm producer (M): 2 × ODc < OD ≤ 4 × ODc; or strong biofilm producer (S): OD > 4 × ODc.

### Statistical analysis

All statistical analyses were conducted in SAS9.2 software (SAS Institute Inc., Cary, NC, USA). All statistical tests were two-sided, and P < 0.05 was considered statistically significant. The chi-square test and Fisher’s exact test were selected to analyse the biofilm formation differences among groups.The Bonferroni method was used to conduct multiple comparisons.

## Additional Information

**How to cite this article**: Hu, Y. *et al*. Biofilm may not be Necessary for the Epidemic Spread of *Acinetobacter baumannii*. *Sci. Rep.* **6**, 32066; doi: 10.1038/srep32066 (2016).

## Supplementary Material

Supplementary Table S1

## Figures and Tables

**Table 1:** Characteristics of the clinical isolates used in this study.

Bacterial species	no. of isolates	no. of hospitals	no. of PFGE types#	Drug resistance*	Site of isolation
*A. baumannii*	114	4	41	MDR (n = 10)	Wound (n = 1)
				XDR (n = 83)	Throat swab (n = 3)
				S (n = 21)	Sputum (n = 55)
					Hydrothorax (n = 1)
					Drainage fluids (n = 1)
					CSF (n = 1)
					Blood (n = 2)
					Ascites (n = 2)
					Unknown (n = 48)
*A. pittii*	13	5	12	MDR (n = 3)	Throat swab (n = 1)
				XDR (n = 2)	Sputum (n = 6)
				S (n = 8)	Unknown (n = 6)
*A. nosocomialis*	8	4	NA	XDR (n = 1)	Wound (n = 1)
				S (n = 7)	Sputum (n = 7)
*A.junii*	7	3	NA	S (n = 7)	Sputum (n = 5)
					Unknown (n = 2)
*A. bereziniae*	4	3	NA	XDR (n = 2)	Sputum (n = 4)
				S (n = 2)	

^#^NA: not performed.

^*^MDR: resistant to at least three classes of antimicrobial agents, including all penicillins and cephalosporins (including inhibitor combinations), fluoroquinolones, and aminoglycosides; XDR: MDR, also resistant to carbapenems; S: non-MDR.

**Table 2:** Comparison of the biofilm formation capacities of clinical *A. baumannii* isolates and non-*baumannii Acinetobacter* isolates (non-AB).

Biofilm formation^a^	*A. baumannii*				non-AB
	outbreak	epidemic	sporadic (R)^b^	sporadic (S)^c^	
N	26	29	13	5	6
W	3	10	4	4	10
M	0	2	4	6	7
S	0	1	1	6	9
SUM (+%*)	**29 (10.3%)**	**42 (31%)**	**22 (41%)**	**21 (76.2%)**	**32 (81.3%)**

Chi-square test: P < 0.0001. The Bonferroni method was used to conduct multiple comparisons. Significant differences were found between outbreak and sporadic (S) (P < 0.0001), outbreak and non-AB (P < 0.0001), epidemic and sporadic (S) (P < 0.0001), and epidemic and non-AB (P < 0.0001).

^a^N: non-biofilm producer, W: weak biofilm producer, M: moderate biofilm producer, S: strong biofilm producer.

^b^MDR sporadic isolates.

^c^Non-MDR sporadic isolates.

^*^The positive rate of biofilm formation for each group.

**Table 3:** Biofilm formation capacities of the clinical *A. baumannii* isolates of each PFGE type.

PFGE type	No. of isolates	Hospital	MLST ST (allelic profile)^a^	Drug resistance*	No. of isolates^b^				Positive rate	OD/ODc range^c^	Epidemicity
					N	W	M	S			
P4	12	BJ	ST2(2-2-2-2-2-2-2)	3 MDR, 9 XDR	9	3			25%	1.01~1.37	epidemic
P7	4	BJ	ST2(2-2-2-2-2-2-2)	XDR	3	1			25%	1.01	epidemic
P12	11	YT	ST2(2-2-2-2-2-2-2)	2 MDR, 9 XDR	7	3	1		36.4%	1.01~3.33	epidemic
P14	8	HN	ST2(2-2-2-2-2-2-2)	XDR	6		1	1	25%	2.84, 13.24	epidemic
P16	7	HN	ST2(2-2-2-2-2-2-2)	XDR	4	3			42.9%	1.01~1.97	epidemic
P10	29	BJ	ST2(2-2-2-2-2-2-2)	1 MDR, 28 XDR	26	3			10.3%	1.07~1.56	outbreak
P1	1	BJ	ST2(2-2-2-2-2-2-2)	XDR		1			100%	1.12	sporadic
P2	1	BJ	ST2(2-2-2-2-2-2-2)	MDR	1				0		sporadic
P3	1	BJ	ST2(2-2-2-2-2-2-2)	XDR	1				0		sporadic
P5	1	YT	ST2(2-2-2-2-2-2-2)	XDR			1		100%	2.51	sporadic
P6	2	BJ	ST2(2-2-2-2-2-2-2)	MDR	1		1		50%	2.24	sporadic
P8	1	HN	ST2(2-2-2-2-2-2-2)	XDR	1				0		sporadic
P9	1	HN	ST2(2-2-2-2-2-2-2)	XDR	1				0		sporadic
P11	1	YT	ST2(2-2-2-2-2-2-2)	XDR	1				0		sporadic
P13	1	BJ	ST2(2-2-2-2-2-2-2)	XDR	1				0		sporadic
P15	2	HN	ST2(2-2-2-2-2-2-2)	XDR	2				0		sporadic
P17	2	HN	ST2(2-2-2-2-2-2-2)	XDR		1	1		100%	1.06, 2.17	sporadic
P18	2	WZ	ST2(2-2-2-2-2-2-2)	XDR	2				0		sporadic
P19	2	WZ	ST2(2-2-2-2-2-2-2)	XDR	1	1			50%	1.89	sporadic
P26	2	YT	ST131(3-2-2-2-3-2-6)	1 MDR, 1 XDR		1		1	100%	1.62, 4.9	sporadic
P21	1	YT	ST376(27-4-2-1-42-1-2)	XDR			1		100%	2.2	sporadic
P34	1	HN	ST46(5-12-11-2-14-9-14)	XDR	1				0		sporadic
P33	1	HN	ST246(1-49-3-4-5-2-36)	S		1			100%	1.1	sporadic
P37	1	YT	ST36(1-2-2-2-3-1-2)	S			1		100%	3.05	sporadic
P24	2	HN	ST131(3-2-2-2-3-2-6)	S			2		100%	3.08, 3.99	sporadic
P25	1	HN	ST131(3-2-2-2-3-2-6)	S			1		100%	2.35	sporadic
P29	1	HN	ST23(1-3-10-1-4-4-4)	S				1	100%	24.08	sporadic
P31	1	BJ	ST40(1-2-2-2-5-1-14)	S				1	100%	5.6	sporadic
P32	1	HN	ST40(1-2-2-2-5-1-14)	S	1				0		sporadic
P36	1	HN	ST40(1-2-2-2-5-1-14)	S				1	100%	5.46	sporadic
P27	1	HN	ST216(1-4-2-2-7-1-2)	S	1				0		sporadic
P28	1	HN	ST203(3-4-2-2-7-1-2)	S	1				0		sporadic
P22	1	HN	ST354(3-2-2-2-7-1-5)	S		1			100%	1.03	sporadic
P23	1	YT	ST193(3-1-7-1-7-2-4)	S	1				0		sporadic
P20	1	HN	ST763(3-4-2-2-9-1-5)	S				1	100%	5.04	sporadic
P38	1	WZ	ST252(1-4-3-2-9-1-5)	S		1			100%	1.31	sporadic
P40	1	BJ	N (1-1-2-3-12-1-5)	S		1			100%	1.18	sporadic
P30	1	WZ	ST338(8-5-2-26-13-1-2)	S			1		100%	2.57	sporadic
P35	1	WZ	ST46(5-12-11-2-14-9-14)	S				1	100%	6.92	sporadic
P39	1	BJ	ST372(1-4-2-1-42-1-2)	S	1				0		sporadic
P41	1	HN	ST372(1-4-2-1-42-1-2)	S			1		100%	2.09	sporadic
P42	1	HN	ST372(1-4-2-1-42-1-2)	S				1	100%	4.72	sporadic
	114				73	21	12	8	36%	1.01~24.08	
				64.0%	18.4%	10.5%	7.0%				

^a^A new ST was revealed, named N in this study.

^b^N: non-biofilm producer, W: weak biofilm producer, M: moderate biofilm producer, S: strong biofilm producer.

^c^mean OD/ODc range for the biofilm-positive isolates, biofilm negative isolates were not included. Single mean OD/ODc values are listed for PFGE types with only one positive isolate.

^*^MDR: resistant to at least three classes of antimicrobial agents, including all penicillins and cephalosporins (including inhibitor combinations), fluoroquinolones, and aminoglycosides; XDR: MDR, also resistant to carbapenems; S: non-MDR.

**Table 4:** Biofilm formation capacities of the clinical *A. baumannii* isolates of each MLST sequence type (ST).

MLST type^a^ST (allelic profile)^a^	No. of isolates	Hospital	PFGE type	Drug resistance#	No. of isolates^b^				Positive rate	OD/ODc ratio range^c^
					N	W	M	S		
ST2(2-2-2-2-2-2-2)	89	BJ, HN, YT, WZ	P1~P19	9MDR, 80XDR	67	16	5	1	24.7%	1.01~13.24
ST131(3-2-2-2-3-2-6)	5	HN, YT	P24~P26	1MDR, 1XDR, 3S		1	3	1	100%	1.62~4.9
ST372(1-4-2-1-42-1-2)	3	BJ, HN	P39, P41, P42	S	1		1	1	66.7%	2.09, 4.72
ST40(1-2-2-2-5-1-14)	3	BJ, HN	P31, P32, P36	S	1			2	66.7%	5.46, 5.6
ST46(5-12-11-2-14-9-14)	2	HN, WZ	P34, P35	XDR, S	1			1	50%	6.92
ST23(1-3-10-1-4-4-4)	1	HN	P29	S				1	100%	24.08
ST763(3-4-2-2-9-1-5)	1	HN	P20	S				1	100%	5.04
ST36(1-2-2-2-3-1-2)	1	YT	P37	S			1		100%	3.05
ST338(8-5-2-26-13-1-2)	1	WZ	P30	S			1		100%	2.57
ST376(27-4-2-1-42-1-2)	1	YT	P21	XDR			1		100%	2.20
ST252(1-4-3-2-9-1-5)	1	WZ	P38	S		1			100%	1.31
N(1-1-2-3-12-1-5)	1	BJ	P40	S		1			100%	1.18
ST246(1-49-3-4-5-2-36)	1	HN	P33	S		1			100%	1.10
ST354(3-2-2-2-7-1-5)	1	HN	P22	S		1			100%	1.03
ST193(3-1-7-1-7-2-4)	1	YT	P23	S	1				0	
ST203(3-4-2-2-7-1-2)	1	HN	P28	S	1				0	
ST216(1-4-2-2-7-1-2)	1	HN	P27	S	1				0	
SUM of non-ST2*	25				6	5	7	7	76%	1.03~24.08

^a^A new ST was revealed, named N in this study.

^b^N: non-biofilm producer, W: weak biofilm producer, M: moderate biofilm producer, S: strong biofilm producer.

^c^Range of the mean OD/ODc for the biofilm-positive isolates. A single mean OD/ODc value was listed for the MLST type with only one positive isolate.

^*^Significant difference was found between IC2 and non-IC2, Fisher’s exact test, P < 0.0001.

^#^MDR: resistant to at least three classes of antimicrobial agents, including all penicillins and cephalosporins (including inhibitor combinations), fluoroquinolones, and aminoglycosides; XDR: MDR, also resistant to carbapenems; S: non-MDR.
