# Supplementary material for: Biofilm may not be Necessary for the Epidemic Spread of Acinetobacter baumannii
Source: Sci Rep. 2016 Aug 25;6:32066. doi: 10.1038/srep32066 (PMC4997352; doi:10.1038/srep32066)
Supplement: Supplementary Table S1 [file srep32066-s1.pdf]

Biofilm may not be Necessary for the Epidemic Spread of *Acinetobacter baumannii*

Yuan Hu, Lihua He, Xiaoxia Tao, Fanliang Meng, Jianzhong Zhang

Supplementary Table S1. Biofilm formations of 146 clinical *Acinetobacter* isolates.

Isolate	Species	Hospital	Source	Drug resistance#	MLST	PFGE	OD/ ODc (ranges)	Biofilm*
**Outbreak *A. baumannii* isolates**								
BJ23	*A. baumannii*	BJ		XDR	ST2	P10	0.65 (0.61, 0.69)	N
BJ74	*A. baumannii*	BJ		XDR	ST2	P10	0.67 (0.61, 0.72)	N
BJ24	*A. baumannii*	BJ		XDR	ST2	P10	0.68 (0.63, 0.75)	N
BJ27	*A. baumannii*	BJ		XDR	ST2	P10	0.68 (0.65, 0.72)	N
BJ10	*A. baumannii*	BJ		XDR	ST2	P10	0.70 (0.62, 0.78)	N
BJ42	*A. baumannii*	BJ		XDR	ST2	P10	0.73 (0.67, 0.80)	N
BJ48	*A. baumannii*	BJ		MDR	ST2	P10	0.74 (0.67, 0.80)	N
BJ21	*A. baumannii*	BJ		XDR	ST2	P10	0.75 (0.66, 0.84)	N
BJ39	*A. baumannii*	BJ		XDR	ST2	P10	0.75 (0.66, 0.94)	N
BJ82	*A. baumannii*	BJ	Drainage fluids	XDR	ST2	P10	0.75 (0.69, 0.84)	N
BJ84	*A. baumannii*	BJ		XDR	ST2	P10	0.76 (0.71, 0.80)	N
BJ44	*A. baumannii*	BJ	Sputum	XDR	ST2	P10	0.79 (0.65, 1.06)	N
BJ81	*A. baumannii*	BJ		XDR	ST2	P10	0.79 (0.66, 0.92)	N
BJ18	*A. baumannii*	BJ		XDR	ST2	P10	0.80 (0.72, 0.94)	N
BJ55	*A. baumannii*	BJ		XDR	ST2	P10	0.80 (0.73, 0.85)	N
BJ9	*A. baumannii*	BJ		XDR	ST2	P10	0.81 (0.58, 0.98)	N
BJ69	*A. baumannii*	BJ		XDR	ST2	P10	0.81 (0.69, 0.89)	N
BJ33	*A. baumannii*	BJ		XDR	ST2	P10	0.82 (0.78, 0.85)	N
BJ51	*A. baumannii*	BJ	Sputum	XDR	ST2	P10	0.82 (0.80, 0.84)	N
BJ34	*A. baumannii*	BJ		XDR	ST2	P10	0.83 (0.70, 0.91)	N
BJ32	*A. baumannii*	BJ		XDR	ST2	P10	0.85 (0.77, 1.00)	N
BJ38	*A. baumannii*	BJ	Ascites	XDR	ST2	P10	0.86 (0.80, 0.92)	N
BJ16	*A. baumannii*	BJ		XDR	ST2	P10	0.87 (0.79, 0.95)	N
BJ47	*A. baumannii*	BJ		XDR	ST2	P10	0.87 (0.82, 0.92)	N
BJ36	*A. baumannii*	BJ		XDR	ST2	P10	0.94 (0.75, 1.25)	N
BJ52	*A. baumannii*	BJ	Sputum	XDR	ST2	P10	0.95 (0.81, 1.08)	N
BJ66	*A. baumannii*	BJ		XDR	ST2	P10	1.07 (1.02, 1.15)	W
BJ35	*A. baumannii*	BJ	Sputum	XDR	ST2	P10	1.18 (0.99, 1.33)	W
BJ83	*A. baumannii*	BJ		XDR	ST2	P10	1.56 (1.28, 2.00)	W
**Epidemic *A.baumannii* isolates**								
YT12	*A. baumannii*	YT	Sputum	XDR	ST2	P12	0.69 (0.65, 0.75)	N
YT8	*A. baumannii*	YT	Sputum	XDR	ST2	P12	0.71 (0.60, 0.81)	N
YT9	*A. baumannii*	YT	Sputum	MDR	ST2	P12	0.73 (0.67, 0.79)	N
YT13	*A. baumannii*	YT	Sputum	MDR	ST2	P12	0.76 (0.71, 0.82)	N
YT5	*A. baumannii*	YT	Sputum	XDR	ST2	P12	0.78 (0.63, 1.02)	N

YT11	*A. baumannii*	YT	wound	XDR	ST2	P12	0.91 (0.89, 0.95)	N
YT20	*A. baumannii*	YT	CSF	XDR	ST2	P12	0.98 (0.87, 1.15)	N
YT16	*A. baumannii*	YT	Sputum	XDR	ST2	P12	1.01 (0.75, 1.31)	W
YT7	*A. baumannii*	YT	Sputum	XDR	ST2	P12	1.01 (0.91, 1.11)	W
YT26	*A. baumannii*	YT	Sputum	XDR	ST2	P12	1.03 (0.80, 1.41)	W
YT4	*A. baumannii*	YT	Sputum	XDR	ST2	P12	3.33 (1.43, 6.78)	M
HN089	*A. baumannii*	HN	Sputum	XDR	ST2	P14	0.69 (0.63, 0.72)	N
HN026	*A. baumannii*	HN	Sputum	XDR	ST2	P14	0.76 (0.70, 0.85)	N
HN256	*A. baumannii*	HN	Sputum	XDR	ST2	P14	0.80 (0.72, 0.87)	N
HN029	*A. baumannii*	HN	Sputum	XDR	ST2	P14	0.80 (0.75, 0.85)	N
HN055	*A. baumannii*	HN	Sputum	XDR	ST2	P14	0.83 (0.79, 0.89)	N
HN031	*A. baumannii*	HN	Sputum	XDR	ST2	P14	0.94 (0.90, 1.00)	N
HN006	*A. baumannii*	HN	Sputum	XDR	ST2	P14	13.24 (6.34,19.5)	S
HN030	*A. baumannii*	HN	Sputum	XDR	ST2	P14	2.84 (1.93, 4.25)	M
HN005	*A. baumannii*	HN	Sputum	XDR	ST2	P16	0.72 (0.67, 0.78)	N
HN002	*A. baumannii*	HN	Sputum	XDR	ST2	P16	0.78 (0.72, 0.85)	N
HN004	*A. baumannii*	HN	Sputum	XDR	ST2	P16	0.86 (0.71,0.99)	N
HN085	*A. baumannii*	HN	Sputum	XDR	ST2	P16	0.86 (0.78, 0.93)	N
HN121	*A. baumannii*	HN	Sputum	XDR	ST2	P16	1.01 (0.81, 1.28)	W
HN001	*A. baumannii*	HN	Sputum	XDR	ST2	P16	1.57 (1.26, 1.81)	W
HN127	*A. baumannii*	HN	Sputum	XDR	ST2	P16	1.97 (1.56, 2.37)	W
BJ87	*A. baumannii*	BJ		XDR	ST2	P4	0.68 (0.65, 0.72)	N
BJ46	*A. baumannii*	BJ		XDR	ST2	P4	0.69 (0.58, 0.89)	N
BJ68	*A. baumannii*	BJ		XDR	ST2	P4	0.70 (0.63, 0.82)	N
BJ86	*A. baumannii*	BJ		XDR	ST2	P4	0.74 (0.68, 0.82)	N
BJ45	*A. baumannii*	BJ		XDR	ST2	P4	0.78 (0.70, 0.90)	N
BJ50	*A. baumannii*	BJ		MDR	ST2	P4	0.82 (0.79, 0.87)	N
BJ20	*A. baumannii*	BJ		XDR	ST2	P4	0.91 (0.84, 1.03)	N
BJ54	*A. baumannii*	BJ		XDR	ST2	P4	0.93 (0.89,0.97)	N
BJ65	*A. baumannii*	BJ		XDR	ST2	P4	1 .00(0.80, 1.36)	N
BJ53	*A. baumannii*	BJ	Sputum	XDR	ST2	P4	1.01 (0.88, 1.13)	W
BJ7	*A. baumannii*	BJ		MDR	ST2	P4	1.24 (0.87, 1.64)	W
BJ19	*A. baumannii*	BJ		MDR	ST2	P4	1.37 (0.90, 2.20)	W
BJ12	*A. baumannii*	BJ		XDR	ST2	P7	0.67 (0.61, 0.72)	N
BJ60	*A. baumannii*	BJ		XDR	ST2	P7	0.71 (0.66, 0.79)	N
BJ22	*A. baumannii*	BJ		XDR	ST2	P7	0.77 (0.71, 0.83)	N
BJ37	*A. baumannii*	BJ	Ascites	XDR	ST2	P7	1.01 (0.91, 1.09)	W
**MDR sporadic *A. baumannii* isolates**								
WZ055	*A. baumannii*	WZ	Sputum	XDR	ST2	P19	0.64 (0.6, 0.68)	N
WZ021	*A. baumannii*	WZ		XDR	ST2	P18	0.68 (0.65, 0.72)	N
HN250	*A. baumannii*	HN	hydrothorax	XDR	ST2	P15	0.70 (0.65, 0.76)	N
HN007	*A. baumannii*	HN	Sputum	XDR	ST2	P8	0.73 (0.62, 0.8)	N
BJ76	*A. baumannii*	BJ		MDR	ST2	P6	0.73 (0.7, 0.79)	N
WZ110	*A. baumannii*	WZ		XDR	ST2	P18	0.76 (0.61, 0.91)	N

BJ31	*A. baumannii*	BJ		XDR	ST2	P3	0.76 (0.61, 1.00)	**N**
BJ25	*A. baumannii*	BJ		XDR	ST2	P13	0.78 (0.65, 1.00)	**N**
YT24	*A. baumannii*	YT	Sputum	XDR	ST2	P11	0.78 (0.69, 0.86)	**N**
HN097	*A. baumannii*	HN	Sputum	XDR	ST2	P9	0.79 (0.70, 0.85)	**N**
HN246	*A. baumannii*	HN	Blood	XDR	ST2	P15	0.83 (0.66, 1.13)	**N**
BJ80	*A. baumannii*	BJ		MDR	ST2	P2	0.83 (0.71, 0.95)	**N**
HN125	*A. baumannii*	HN	Sputum	XDR	ST46	P34	0.95 (0.78, 1.25)	**N**
HN028	*A. baumannii*	HN	Sputum	XDR	ST2	P17	1.06 (0.93, 1.27)	**W**
BJ79	*A. baumannii*	BJ		XDR	ST2	P1	1.12 (0.95, 1.29)	**W**
YT15	*A. baumannii*	YT	Sputum	XDR	ST131	P26	1.62 (0.88,2.89)	**W**
WZ057	*A. baumannii*	WZ	Sputum	XDR	ST2	P19	1.89 (1.56, 2.35)	**W**
HN027	*A. baumannii*	HN	Sputum	XDR	ST2	P17	2.17 (1.20,2.72)	**M**
YT10	*A. baumannii*	YT	Sputum	XDR	ST376	P21	2.20 (1.84, 2.82)	**M**
BJ4	*A. baumannii*	BJ		MDR	ST2	P6	2.24 (1.28, 3.19)	**M**
YT21	*A. baumannii*	YT	Sputum	XDR	ST2	P5	2.51 (1.54,3.47)	**M**
YT2	*A. baumannii*	YT	Sputum	MDR	ST131	P26	4.90 (2.29,7.50)	**S**
**Non-MDR sporadic *A. baumannii* isolates**								
HN124	*A. baumannii*	HN	Sputum	S	ST216	P27	0.74 (0.64, 0.82)	**N**
YT3	*A. baumannii*	YT	Blood	S	ST193	P23	0.76 (0.69, 0.86)	**N**
HN251	*A. baumannii*	HN	Sputum	S	ST203	P28	0.77 (0.70, 0.83)	**N**
BJ6	*A. baumannii*	BJ		S	ST372	P39	0.92 (0.80, 1.05)	**N**
HN043	*A. baumannii*	HN	Sputum	S	ST40	P32	0.93 (0.83, 1.04)	**N**
HN086	*A. baumannii*	HN	Sputum	S	ST354	P22	1.03 (0.85, 1.28)	**W**
HN165	*A. baumannii*	HN	Sputum	S	ST246	P33	1.10 (0.92, 1.25)	**W**
BJ70	*A. baumannii*	BJ		S	1-1-2-3-12-1-5	P40	1.18 (1.11, 1.25)	**W**
WZ072	*A. baumannii*	WZ	Sputum	S	ST252	P38	1.31 (1.07, 1.56)	**W**
HN253	*A. baumannii*	HN	Throat swab	S	ST372	P41	2.09 (1.55, 3.00)	**M**
HN090	*A. baumannii*	HN	Sputum	S	ST131	P25	2.35 (1.56, 3.49)	**M**
WZ068	*A. baumannii*	WZ	Sputum	S	ST338	P30	2.57 (1.62, 4.30)	**M**
HN094	*A. baumannii*	HN	Sputum	S	ST23	P29	24.08 (13.6, 31.53)	**S**
YT25	*A. baumannii*	YT	Sputum	S	ST36	P37	3.05 (2.46,3.87)	**M**
HN051	*A. baumannii*	HN	Sputum	S	ST131	P24	3.08 (1.88, 4.35)	**M**
HN049	*A. baumannii*	HN	Throat swab	S	ST131	P24	3.99 (2.55, 6.31)	**M**
HN052	*A. baumannii*	HN	Throat swab	S	ST372	P42	4.72 (3.70, 6.57)	**S**
HN245	*A. baumannii*	HN	Sputum	S	ST763	P20	5.04 (4.41, 6.03)	**S**
HN164	*A. baumannii*	HN	Sputum	S	ST40	P36	5.46 (4.83, 6.06)	**S**
BJ17	*A. baumannii*	BJ		S	ST40	P31	5.60 (4.21, 6.32)	**S**
WZ173	*A. baumannii*	WZ	Sputum	S	ST46	P35	6.92 (5.40, 8.07)	**S**
**non-*baumannii Acinetobacter* isolates**								
HBXH129	*A.junii*	HB	Sputum	S			0.78 (0.68, 0.90)	**N**
HN162	*A. nosocomialis*	HN	Sputum	S			0.81 (0.74, 0.9)	**N**
S13041	*A. pittii*	RM		S			0.84 (0.74, 0.97)	**N**
HBXH135	*A. pittii*	HB	Sputum	S			0.85 (0.78, 0.91)	**N**

S12236	*A.junii*	RM		S	0.88 (0.70, 1.05)	**N**
HBXH83	*A. pittii*	HB	Sputum	S	0.90 (0.70, 1.15)	**N**
S12316	*A.junii*	RM		S	1.01 (0.96, 1.07)	**W**
BDYY64	*A.junii*	BD	Sputum	S	1.09 (0.91, 1.29)	**W**
LBQ1	*A. pittii*	YT	Sputum	XDR	1.21 (1.01, 1.27)	**W**
S12192	*A. pittii*	RM		S	1.28 (1.09, 1.63)	**W**
LBQ14	*A. pittii*	YT	Sputum	MDR	1.32 (1.19, 1.52)	**W**
W053	*A. nosocomialis*	WZ	Sputum	S	1.46 (1.17, 1.95)	**W**
S12383	*A. pittii*	RM		S	1.61 (1.39, 1.87)	**W**
HBXH146	*A.junii*	HB	Sputum	S	1.71 (1.33,2.08)	**W**
HN252	*A. nosocomialis*	HN	Sputum	S	1.87 (1.52, 2.16)	**W**
BJ73	*A. pittii*	BJ		S	1.89 (1.59, 2.30)	**W**
LBQ6	*A. pittii*	YT	Sputum	MDR	12.66 (7.21,19.98)	**S**
R6	*A. nosocomialis*	RM	Wound	XDR	14.44 (8.88,20.09)	**S**
R12	*A. bereziniae*	RM	Sputum	XDR	16.99 (14.07,20.61)	**S**
R11	*A. bereziniae*	RM	Sputum	XDR	19.53 (13.36,25.7)	**S**
W060	*A. nosocomialis*	WZ	Sputum	S	2.06 (0.99, 4.16)	**M**
HN145	*A. nosocomialis*	HN	Sputum	S	2.51 (2.06, 2.85)	**M**
HN128	*A. bereziniae*	HN	Sputum	S	2.55 (2.25, 3.05)	**M**
LBQ23	*A. pittii*	YT	Sputum	MDR	3.08 (1.98, 4.18)	**M**
FZ1	*A. nosocomialis*	FZ	Sputum	S	3.24 (1.60, 4.56)	**M**
HBXH72	*A. bereziniae*	HB	Sputum	S	3.43 (3.14, 3.85)	**M**
BDYY1	*A.junii*	BD	Sputum	S	3.49 (2.52, 4.45)	**M**
H015	*A. pittii*	TZ		S	4.25 (2.50, 5.39)	**S**
R18	*A. pittii*	RM	Throat swab	XDR	5.02 (3.91,6.13)	**S**
HBXH110	*A.junii*	HB	Sputum	S	5.38 (2.60, 7.89)	**S**
S12159	*A. pittii*	RM		S	5.41 (4.74, 6.22)	**S**
HN095	*A. nosocomialis*	HN	Sputum	S	6.30 (5.53, 7.10)	**S**

#. MDR: resistant to at least three classes of antimicrobial agents, including all penicillins and cephalosporins (including inhibitor combinations), fluoroquinolones, and aminoglycosides; XDR: MDR, also resistant to carbapenems; S: non-MDR.

*. N: non-biofilm, W: weak biofilm, S: strong biofilm.
